# Development of a Risk Nomogram Model for Identifying Interstitial Lung Disease in Patients With Rheumatoid Arthritis

**DOI:** 10.3389/fimmu.2022.823669

**Published:** 2022-06-16

**Authors:** Jing Xue, Wenfeng Hu, Shuang Wu, Jing Wang, Shuhong Chi, Xiaoming Liu

**Affiliations:** ^1^ Key Laboratory of Ministry of Education for Conservation and Utilization of Special Biological Resources in the Western, College of Life Science, Ningxia University, Yinchuan, China; ^2^ Ningxia Key Laboratory of Stem Cell and Regenerative Medicine, General Hospital of Ningxia Medical University, Yinchuan, China; ^3^ Department of Pathology, General Hospital of Ningxia Medical University, Yinchuan, China; ^4^ Department of Rheumatology, General Hospital of Ningxia Medical University, Yinchuan, China; ^5^ Department of Anatomy and Cell Biology, Carver College of Medicine, the University of Iowa, Iowa City, IA, United States

**Keywords:** nomogram, rheumatoid arthritis, interstitial lung disease, matrix metalloproteinase-3, risk factors

## Abstract

The clinical features of rheumatoid arthritis (RA)-associated interstitial lung disease (ILD) (RA-ILD) usually manifest to an advanced stage of lung disease, which leads the challenge of early diagnosis and the difficulty in guiding treatments for patients with RA-ILD in clinical settings. The aim of this study was to construct a nomogram for identifying ILD in RA patients. Through the incorporation of the level of matrix metalloproteinase-3 (MMP-3) in plasma, demographics, clinical feature, and laboratory parameters of 223 RA patients (85 RA-ILD) which were grouped as training cohorts and validation cohorts, an identifying nomogram of RA-ILD was built. Candidate variables for the nomogram were screened using univariable analysis and multivariable logistic regression analysis. The accuracy of the diagnostic nomogram was measured *via* concordance index (C-index), calibration plots, and decision curve analysis (DCA). Results showed that plasma MMP-3 protein was elevated in RA-ILD patients compared with non-ILD RA patients in both training cohorts (p = 0.0475) and validation cohorts (p = 0.0006). Following a final regression analysis, the gender of male, current smoking state, levels of circulating rheumatoid factor (RF), C-reactive protein (CRP), and MMP-3 were identified as risk factors for the construction of the nomogram. The calibration plots further showed a favorable consistency between the identifying nomogram and actual clinical findings. In consistence, the C-index (0.826 for both training cohorts and validation cohorts) indicated the satisfactory discriminative ability of the nomogram. Although the incorporation of MMP-3 failed to significantly improve identified outcomes of the nomogram as determined by DCA, including the level of circulating MMP-3 increased the diagnostic accuracy of the nomogram for ILD in RA patients. Thus, our proposed model can serve as a non-invasive tool to identify ILD in RA patients, which may assist physicians to make treatment decisions for RA patients.

## Introduction

Pulmonary involvement is one of common extra-articular manifestations in patients with rheumatoid arthritis (RA) (1, 2), which manifests different clinical phenotypes in RA patients, including pleura disorders, interstitial lung disease (ILD), and obliterative bronchiolitis (OB). Of these, ILD contributes significantly to the morbidity and mortality in RA patients (3, 4). The difficulty in accurate and early diagnosis results in a significant impact on the treatment and prognosis of this disease in clinical settings.

In general, a number of factors can increase the likelihood of progression and mortality in RA-ILD. For instance, the incidence of RA-ILD in men is greater compared to female patients (3, 5). Cigarette smoking is also associated with the development of RA-ILD, as cigarette smoke inhalation induces genotoxic and inflammatory citrullination of lung antigens that extend from the conducting airway to the gas exchange zones, particularly in those patients with the shared human leukocyte antigen DRB1 (HLA-DRB1) epitope (6). The strong interactions between risk factors from environments, genetics, and/or clinics of RA thus reinforced the development of ILD in RA patients.

Consistent with the above paradigm, several groups have shown an increased level of the anti-CCP antibody in patients with RA-ILD (7–10). Beyond the elevated anti-CCP antibody and rheumatoid factor (RF), other alternative biomarkers have also been evaluated for their ability to diagnose RA-ILD, such as the epithelial cell-derived Krebs von den Lungen-6 (KL-6) (11), surfactant protein D (SP-D), matrix metalloproteinase-7 (MMP-7), interferon-γ-inducible protein 10 (IP10/CXCL10), and pulmonary and activation-regulated chemokine (PARC) (12, 13). In addition, several studies on the potential adverse effects of medication treatments with various disease-modifying antirheumatic drugs in RA-ILD development recently gained large attention. Of these agents, methotrexate (MTX) and tumor necrosis factor-alpha inhibitors (TNFi) have been linked to drug-induced pneumonitis, implying their safety issue in RA treatments (14). However, pulmonary involvements particularly ILDs are often unrecognized in RA patients, highlighting a need of clinical tools such as predicting models that are able to identify the early stages of RA-ILD in clinical settings.

RA is a common inflammatory disease, which predominantly degrades articular cartilage. A compelling body of studies demonstrated that cartilage degradation was mediated by enzyme matrix metalloproteinase (MMP) family members. Among these members, MMP-3 (stromelysin-1) is a proteinase synthesized and secreted by synovial fibroblasts, which can result in the degradation of many components of matrix proteins, such as fibronectin, gelatins, and collagens, in the synovial joints. In addition, MMP-3 can also activate other MMPs such as MMP-1, MMP-7, and MMP-9, rendering a crucial role of MMP-3 in connective tissue remodeling (15, 16). A recent study found that the level of circulating MMP-3 was correlated with RA disease activity (17). Of note, further studies revealed that the MMP-3 level was mainly elevated in bronchial and alveolar epithelial cells, interstitial fibroblasts, alveolar macrophages, and other leukocytes of idiopathic pulmonary fibrosis (IPF) lungs (18, 19). Mechanistically, it has been described that MMP3 played a role in fibrogenesis by driving epithelial-to-mesenchymal transition (EMT) (18). These studies strongly support the notion of involvements of MMP-3 in the development of RA and pulmonary fibrosis.

In recent years, the nomogram has been widely used as a predictive approach across the majority of cancer types (20, 21), primarily owing to its ability to meet requirements for an integrated model (22). The practices of prognosis and risk prediction of diseases *via* web have contributed to their popularity among clinicians and patients themselves (22, 23). In light of aforementioned findings of MMP-3 in RA and pulmonary fibrosis, we thus sought to develop a model that incorporated plasma MMP-3 and independent clinical risk factors for identifying ILD in RA patients.

## Methods and Materials

### Study Population and Design

In accordance with protocols approved by the Ethics Committee for the Conduct of Human Research at the General Hospital of Ningxia Medical University (2020-916), blood samples were collected from the outpatient rheumatology and respiratory clinic of the General Hospital of Ningxia Medical University between December 2019 and January 2021. Two hundred twenty-three patients who were diagnosed with RA by fulfilling the 1987 American College of Rheumatology (ACR) classification criteria (24) (25) were further classified into 138 non-ILD RA and 85 RA-ILD based on their chest high-resolution computed tomography (HRCT) and clinical manifestations. A multidisciplinary team (MDT) including pneumologists, radiologists, pathologists, and rheumatologists comprehensively assessed the absence/presence of RA-ILD. Patients with other chronic pulmonary diseases and infectious diseases were excluded. Furthermore, patients who had severe heart, renal, and lung dysfunction were also excluded from this study. Eligible patients were randomly assigned into the training cohort group and validation cohort group. The training cohorts were used to screen risk factors and construct the model. The validation cohorts were used to validate the accuracy of the model generated with training cohorts.

### Microarray Data and Identification of Hub Genes

The transcription profile datasets of RA-fibroblast-like synoviocytes (FLS) (GSE128813) and lung tissue in ILD (GSE47460) were obtained from NCBI GEO databases (http://www.ncbi.nlm.nih.gov/geo/). The platform for GSE128813 is GPL21827, which includes RA-FLS (n = 3) and healthy control groups (n = 3). The platforms for GSE47460 are GPL6480 and GPL14550, which included 582 subjects, of which 254 had ILD, 220 were COPD patients, and 108 were healthy controls. The limma R package (http://www.bioconductor.org/packages/release/bioc/html/limma.html) in Bioconductor was used to identify DEGs by comparing expression values in lung tissue between ILD and healthy control, and FLS between RA and healthy control. The screening criteria were adjusted p < 0.05 and log2 fold change (FC) >1. Based on the STRING database (http://string-db.org), PPIs of DEGs were selected with a filter condition (median confidence>0.4). Next, the PPI network was visualized by Cytoscape Software (http://www.cytoscape.org/). Cytoscape Minimal Common Oncology Data Elements (MCODE) plug-in was applied to find closely connected nodes from the PPI network complex. The resulting PPI network was subjected to module analyses with the Plugin MCODE with the default parameters (degree cutoff ≥2, node score cutoff ≥2, K-core ≥2, and Max depth = 100).

### MMP-3 Measurement

The level of plasma MMP-3 was determined by commercially available ELISA according to the kit’s protocol (NeoBioscience Inc., Shenzhen, China) (assay range: 0.0313–2 ng/ml). For detection of MMP-3 protein, the optical density was measured using the wavelength of 450 nm. Experiments were repeated for three times.

### Demographic, Clinical, Treatment, and Serological Data

Clinical characteristics (age, sex, and current smoking status), laboratory data [rheumatoid factors (RF), erythrocyte sedimentation rate (ESR), C-reactive protein (CRP), and anti-keratin antibody (AKA), lactate dehydrogenase (LDH)], clinical features [RA duration, 28-joint Disease Activity Score (DAS28), swollen joint count, and arthralgic count], and current medication were abstracted from medical records and/or telephone interviews, respectively.

### Statistical Analysis

The independent risk factors of the presence of ILD in RA patients were evaluated using univariate logistic regression analysis in training cohorts. The variables with p-value less than 0.05 were candidates for stepwise multivariate analysis. A nomogram was then established based on the results from the final regression analysis and by using the rms package of R, version 4.1.0 (http://www.r-project.org/). The predictive performance of the nomogram was assessed by concordance index (C-index) and calibration with 1,000 bootstrap samples to decrease the overfit bias and decision curve analysis (DCA) (26, 27). DCA was applied to the nomogram by quantifying net benefits at different threshold probabilities (28). In this study, the clinical benefits of the nomogram constructed with an incorporation of plasma MMP-3 as a variable risk factor over a nomogram without MMP-3 data were compared.

Continuous variables were expressed as mean (SE) and compared with two sample *t-*testing or Mann–Whitney testing. Categorical demographic data were compared using the χ^2^ test or Fisher’s exact test. In all analyses, p < 0.05 was considered statistically significant. All statistical analyses were performed using SPSS for Windows (version 26.0) (SPSS Inc., Chicago, IL, USA) and “rms” and “dca.r” packages in R.

## Results

### Baseline Demographic and Clinical Features

**Figure 1:**
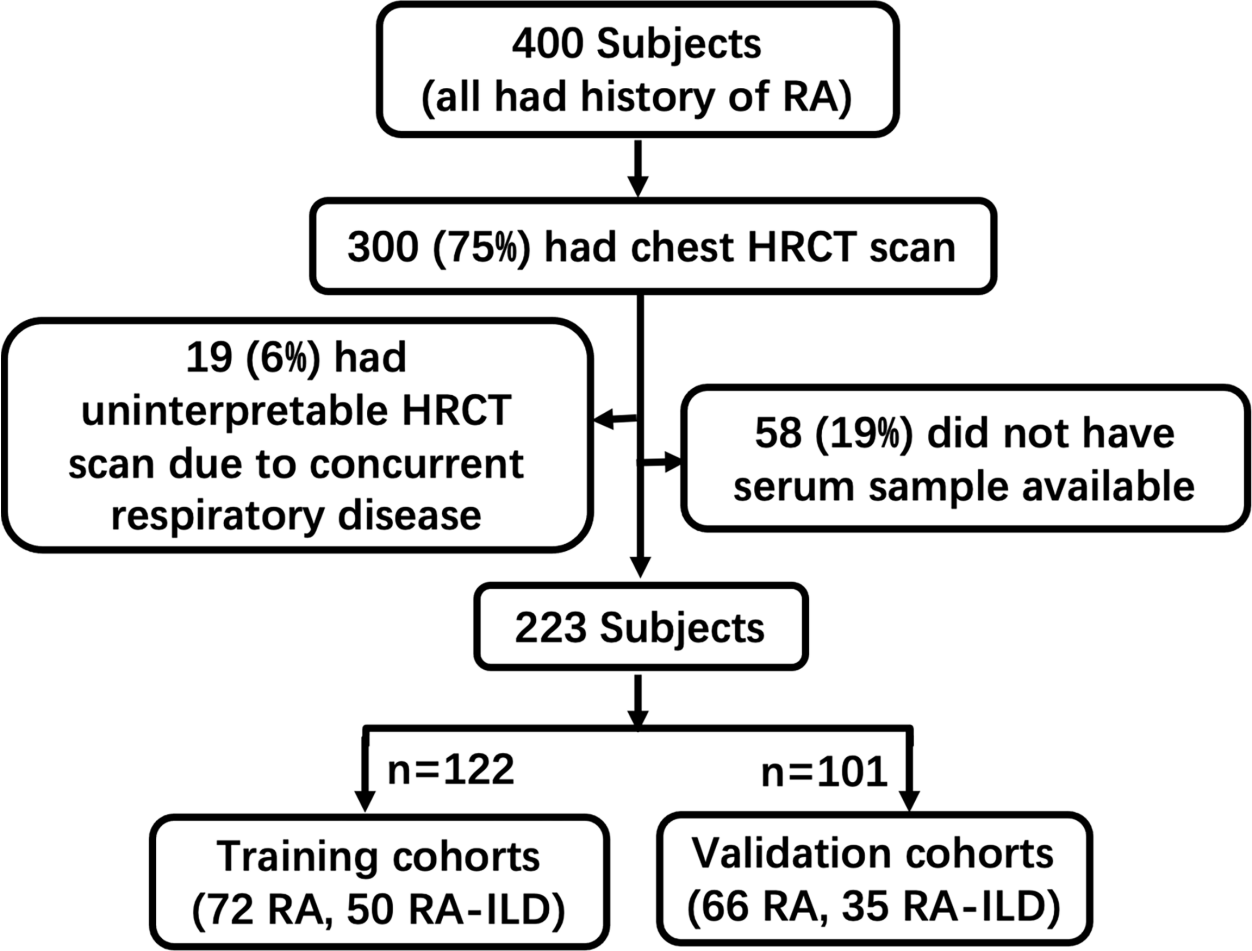
Workflow of sample collection in this study. Patients enrolled in this study were categorized by clinical diagnostic data and HRCT patterns in this study.

A total of 223 patients were enrolled in the study; 100 patients who had no chest HRCT scan, 19 patients who had an uninterpretable HRCT scan, and 58 patients who did not have serum samples available were excluded. Of these, 122 and 101 of them were randomly grouped into the training and validation cohorts according to the timeline, respectively (**Figure 1**). **Table 1** depicts the baseline clinical characteristics of the patients. With respect to baseline clinical data, there was no obvious statistical significance between the training and validation cohorts. In addition, an HRCT-identified ILD was found in 50 (41.0%) and 35 (34.7%) patients in the training and validation cohorts, respectively. Thirty out of 50 (76.0%) patients with imaging features of ILD were UIP, 6 of 50 (12.0%) cases were NSIP, 2 of 50 (4.0%) were OP, and the rest 4 (8.0%) were other ILD patterns in the training cohorts. Twenty-five out of 35 (71.4%) patients with imaging features of ILD were UIP, 2 of 35 (5.7%) cases were NSIP, 5 of 35 (14.3%) were OP, and the rest 3 (8.0%) were other ILD patterns in the validation cohorts.

**Table 1:** Baseline demographic and clinical features of training and validation cohorts.

Variable	Cohorts, no. (%)		*p* value
	Training (n = 122)	Validation (n = 101)	
Demographic features			
Age	51.9 (1.4)	52.2 (1.6)	0.54
Sex			
Male	60 (49.2)	42 (41.6)	0.25
Female	62 (50.8)	59 (58.4)	
Current smoker	56 (46.0)	47 (46.5)	0.41
**Clinical features**			
RA duration, year	7.1 (0.5)	8.0 (0.6)	0.35
Swollen joint count	8 (0.4)	9 (0.4)	0.16
Arthralgic count	7 (0.4)	8 (0.4)	0.11
DAS28 score	3.3 (0.2)	3.4 (0.2)	0.61
**Current medications at baseline**			
NSAIDs	25 (20.5)	21 (20.8)	0.95
Glucocorticoids	28 (23.0)	35 (34.7)	0.05
MTX	27 (22.1)	22 (21.8)	0.95
DMARDs including MTX	69 (56.6)	45 (44.6)	0.07
**Serologic parameters**			
AKA (+)	41 (33.6)	32 (31.7)	0.76
RF (IU/ml)	289.1 (22.2)	276.9 (26.1)	0.72
RF (+)	122 (100.0)	98 (97.0)	0.06
Anti-CCP (+)	50 (41.0)	47 (46.5)	0.40
ESR (mm/h)	92.4 (7.2)	75.6 (5.9)	0.08
CRP (mg/dl)	69.1 (6.0)	57.9 (5.4)	0.18
LDH (U/l)	263.1 (19.2)	275.2 (21.7)	0.68
MMP-3 (ng/ml)	0.43 (0.05)	0.38 (0.02)	0.41
**ILD state**			
Presence	50 (41.0)	35 (34.7)	0.33
Absence	72 (59.0)	66 (65.3)	
**ILD patterns**			
UIP	38 (76.0)	25 (71.4)	0.64
NSIP	6 (12.0)	2 (5.7)	0.32
OP	2 (4.0)	5 (14.3)	0.09
Other	4 (8.0)	3 (8.6)	0.92

Data represented the mean (SE) analyzed by Student t-test using SPSS.

RF, rheumatoid factor; CCP, cyclic citrullinated; ESR, erythrocyte sedimentation rate; CRP, C-reactive protein; LDH, lactic dehydrogenase.

### Elevated MMP-3 Protein in Plasmas of RA-ILD Patients

**Figure 2:**
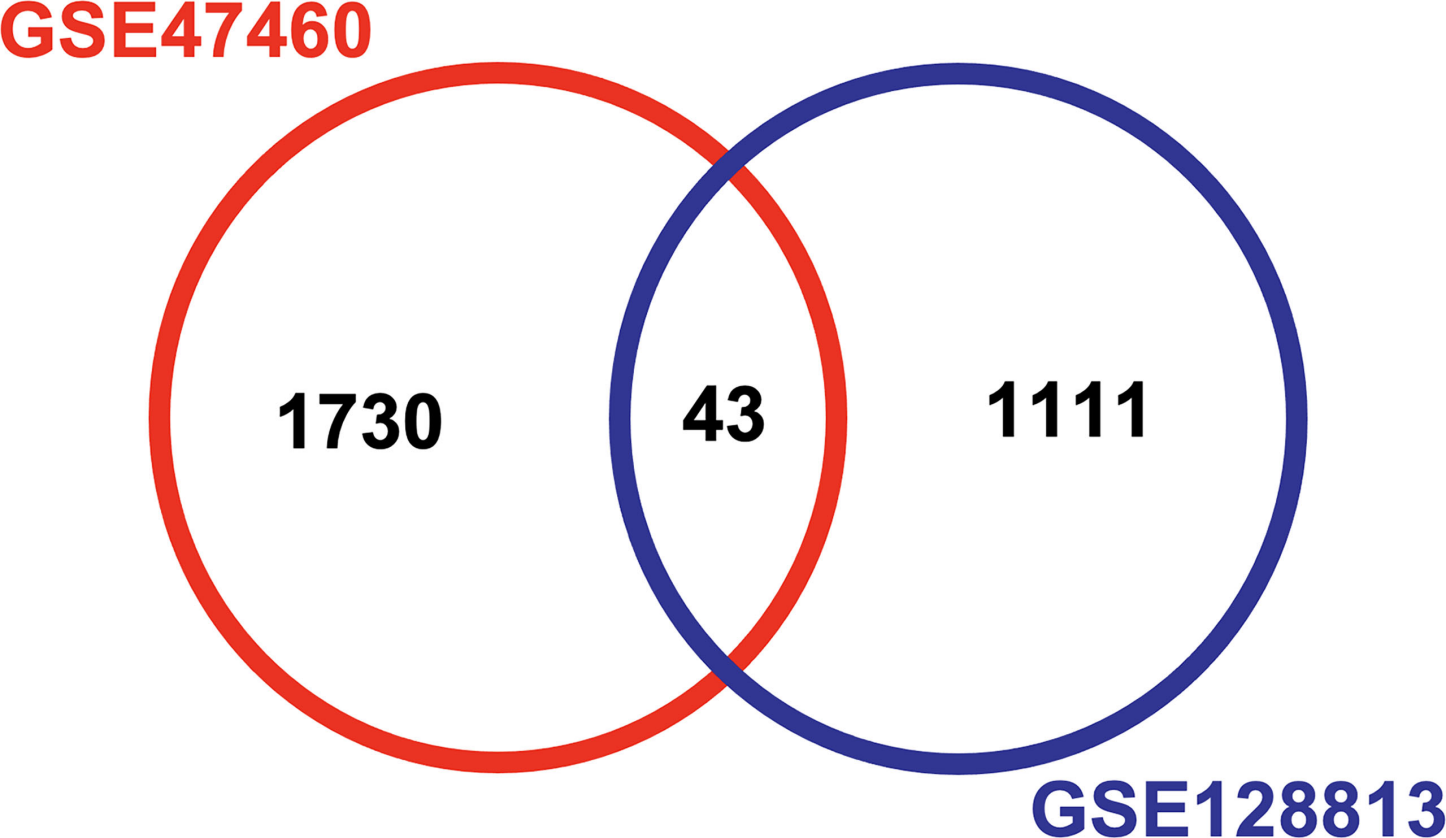
Analysis of differentially expressed genes (DEGs) from GSE128813 and GSE47460 by Venn diagram. The DEGs between fibroblast-like synoviocytes and lung tissue in ILD were identified by two datasets of transcription profiles using Bioinformatics & Evolutionary Genomics (http://bioinformatics.psb.ugent.be/webtools/Venn/). Difference analysis was defined with p < 0.05 and log2 fold change >1 as the cutoff criterion for screening DEGs. A total of expressed 43 DEGs were identified in two datasets. GSE, gene expression series; ILD, interstitial lung disease.

**Figure 3:**
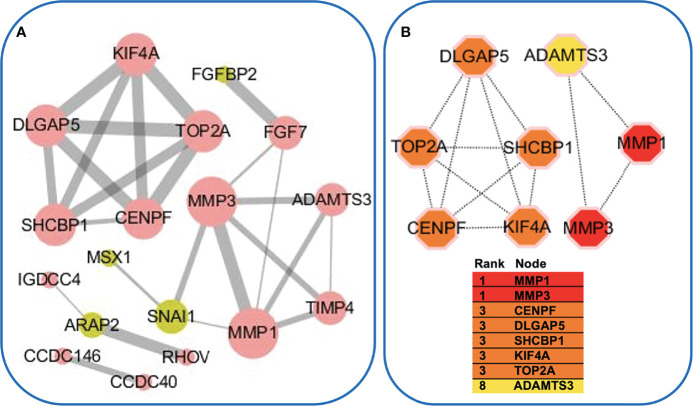
Protein–protein interaction (PPI) network. **(A)** PPI enrichment analysis of differentially expressed genes (DEGs). The nodes denoted proteins, and the edges denoted interactions. The red and green circles represented the up- and down-regulated proteins encoded by DEGs, respectively. The node size and edge width reflected the degree of connectivity. **(B)** Subnetwork of eight hub genes from the PPI network. Node color reflected the degree of connectivity (red to yellow color represent a higher degree to a lower degree).

**Figure 4:**
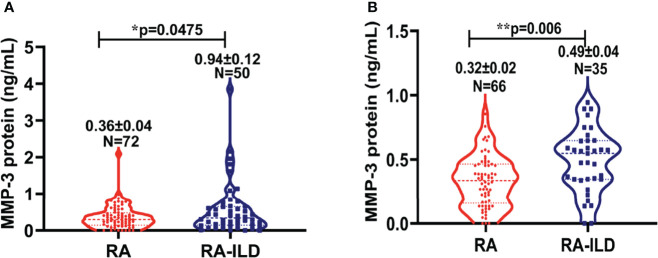
Plasma concentration of MMP-3 protein in RA versus RA-ILD patients. **(A)** The plasma level of MMP-3 protein in RA as well as RA-ILD patients in training cohorts. The level of MMP-3 protein was shown in plasma of RA and RA-ILD patients. The plasma MMP-3 level in RA-ILD was significantly increased compared to in RA control subjects (p = 0.0475). **(B)** The plasma level of MMP-3 protein in RA as well as RA-ILD in validation cohorts. The level of MMP-3 protein was shown in plasma of RA-ILD and non-ILD RA patients. The plasma MMP-3 level in RA-ILD was significantly increased relative to RA patients without ILD (p = 0.006). Bars indicate average protein levels in each group. *p < 0.05; **p < 0.01. Data represent the mean ± SEM in each group.

The GEO databases have been used by many research communities, which can provide an invaluable gene expression profile to derive a new hypothesis (29). We next evaluated the data available in NCBI GEO profiles (GSE128813 and GSE47460) for DEGs from RA-FLS and lung tissue in ILD. A total of the expressed 43 DEGs were identified in two datasets, consisting of 24 up-regulated genes and 19 down-regulated genes (**Figure 2** and **Table 2**). To obtain the best-fit RA-ILD proteins, we filtered these DEGs through the PPI network and visualized them by Cytoscape. As shown in **Figure 3A**, the network consisted of 18 nodes and 25 edges. The red and green circles presented the up-regulated proteins and down-regulated proteins encoded by DEGs, respectively. The top eight hub genes selected by the MCODE in the CytoHubba plug-in included MMP-1, MMP-3, disc large-associated protein 5 (DLGAP5), DNA topoisomerase II alpha (TOP2A), centromere protein F (CENPF), kinesin family member 4A (KIF4A), SHC SH2 domain-binding protein (SHCBP1), and a disintegrin and metalloproteinase with thrombospondin motif (ADAMTS) (**Figure 3B**). Next, we explored plasma MMP-3 in RA patients with and without ILD. The plasma level of the MMP-3 protein significantly increased in RA-ILD patients (0.94 ± 0.12 ng/ml) as compared with RA patients without ILD (0.36 ± 0.04 ng/ml) (p = 0.0457) in the training cohorts (**Figure 4A**). In addition, the concentration of plasma MMP-3 in RA-ILD (0.49 ± 0.04 ng/ml) was also higher compared to RA patients without ILD (0.32 ± 0.02 ng/ml) (p = 0.006) in the validation cohorts (**Figure 4B**).

**Table 2:** Differentially expressed genes from the two datasets.

DEGs	Gene name
Up-regulated (n = 24)	ADAMTS3 AKR1B10 ARAP2 BEX1 CAMP CD93 CENPF DLGAP5 FAM81A FGF14 FGFBP2 GDF6 GFOD1 IGDCC4 IP6K3 KIF4A MMP1 MMP3 MXRA5 SHCBP1 TDO2 TIMP4 TOP2A WFDC12
Down-regulated (n = 19)	ANGPTL7 CCDC146 CCDC40 CD180 FGF7 FRAS1 FRY GBA3 MSX1 PDK4 PHACTR1 PRPH RASD1 RHOV RPS4Y2 SNAI1 SSTR1 SUSD2 UGT2B15

### Nomogram Variable Screening

Following univariable analysis, the variables sex (male) (4.387 [2.020–9.525], p = 0.000), smoking state (current smoker) (5.667 [2.572–12.486], p = 0.000), RF (1.001 [1.000–1.003], p = 0.049), AKA (0.395 [0.174–0.894], p = 0.026), CRP (1.009 [1.002–1.015], p = 0.012), and MMP-3 (2.084 [0.913–4.756], p = 0.043) were incorporated into the multivariable logistic regression analysis. Results demonstrated that the occurrence of ILD was significantly correlated with sex (3.527 [1.325–9.632], p = 0.012), current smoker (3.168 [1.217–8.243], p = 0.018), and circulating concentrations of RF (1.002 [1.000–1.004], p = 0.017), CRP (1.009 [1.002–1.016], p = 0.012), and MMP-3 (2.976 [1.142–7.710], p = 0.026). Male, smoking, circulating RF, CRP, and MMP-3 were risk factors in the development of ILD in RA patients (**Table 3**).

**Table 3:** Univariate and multivariate logistic regression on variables for the prediction of ILD in RA patients.

Characteristic	Univariable		Multivariable	
	HR (95% CI)	*p* value	HR (95% CI)	*p* value
Age	1.019 (0.996–1.043)	0.114		
Sex	4.387 (2.020–9.525)	0.000	3.572 (1.325–9.632)	0.012
Current smoker	5.667 (2.572–12.486)	0.000	3.168 (1.217–8.243)	0.018
RA duration	1.007 (0.947–1.070)	0.832		
Swollen joint count	0.980 (0.903–1.063)	0.625		
Arthralgic count	0.989 (0.918–1.066)	0.799		
DAS28	1.006 (0.821–1.232)	0.953		
NSAIDs	0.950 (0.388–2.328)	0.911		
Glucocorticoids	1.611 (0.689–3.768)	0.271		
MTX	0.659 (0.268–1.615)	0.382		
DMARDs including MTX	0.839 (0.405–1.735)	0.635		
AKA	0.395 (0.174–0.894)	0.026		
RF	1.001 (1.000–1.003)	0.049	1.002 (1.000–1.004)	0.017
Anti-CCP	1.420 (0.682–2.954)	0.349		
ESR	1.001 (0.997–1.006)	0.538		
CRP	1.009 (1.002–1.015)	0.007	1.009 (1.002–1.016)	0.012
LDH	1.001 (0.999–1.003)	0.290		
MMP-3	2.084 (0.913–4.756)	0.043	2.976 (1.142–7.710)	0.026

### Development and Validation of an ILD-Identifying Nomogram

**Figure 5:**
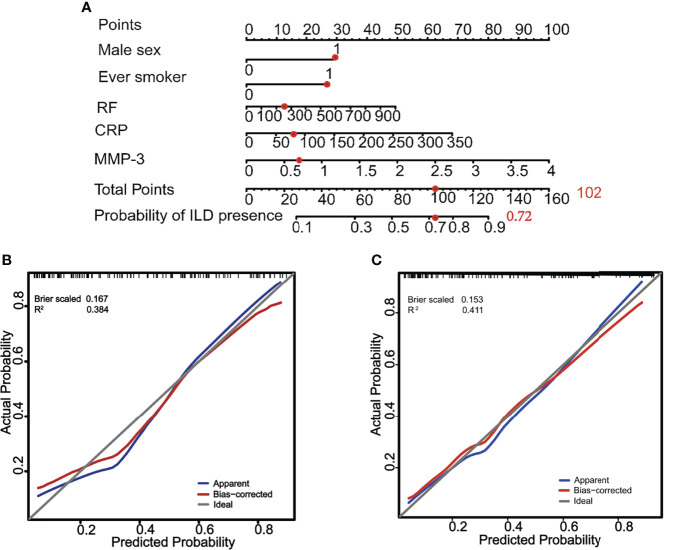
A constructed nomogram for identification of ILD and its diagnostic performance in RA patients. **(A)** a nomogram to estimate the risk of ILD presence of a RA patient (male, smoker, RF = 256 IU/ml, CRP = 82 mg/dl, and MMP-3 = 0.66 ng/ml). To use the nomogram, the sum [30 (male) + 28 (smoker) + 12 (RF) + 14 (CRP) + 18 (MMP-3) = 102] of these points is located on the Total Points axis, and a red dot is drawn downward to the probability of the ILD presence axis to determine the presence (72%) of ILD. **(B)** The calibration curve of probability of ILD presence for RA patients in training cohorts. The x-axis and y-axis represent the nomogram-predicted probability and the actual probability of ILD, respectively. Perfect prediction would correspond to the slope of gray line that makes a 45° angle. The blue line represents all cohorts (n = 122), and the red line expresses bias correction by bootstrapping (B = 1,000 repetitions), indicting the perfect agreement between the presence of ILD and the risk prediction by the nomogram. **(C)** The calibration curve of the probability of ILD presence for RA patients in validation cohorts. The x-axis and y-axis represent the nomogram-predicted probability and the actual probability of ILD, respectively. Perfect prediction would correspond to the gray line with a 45° angle. The blue line represents all cohorts (n = 101), and the red line expresses bias correction by bootstrapping (B = 1,000 repetitions), indicting the perfect agreement between the presence of ILD and the risk prediction by the nomogram.

Based on the variables screened, a nomogram was constructed by incorporating the five significant risk factors for identifying ILD in RA patients. The total nomogram score was determined by the individual scores and applied to obtain the probability for identifying the presence of ILD; most patients had total risk points ranging from 56 to 160 in the present study. While the total risk points were 26, 58, 77, 97, 109, and 128, the presence of ILD were 10%, 30%, 50%, 70%, 80%, and 90%. For instance, a given patient was identified an ILD probability by using the nomogram (**Figure 5A**). Of note, the calibration plots graphically showed good agreement between the identified probability of ILD and the actual observation in the training cohorts and validation cohorts, which indicates good calibration of this model (**Figures 5B, C**). These results suggest that the nomogram built with a combination of variable factors MMP-3, sex, current smoker, RF, and CRP had considerable discriminative and calibrating abilities for identifying ILD in RA patients.

### The Incorporation of Plasma MMP-3 Increases the Identifying Probability of Nomogram for ILD in RA Patients

**Figure 6:**
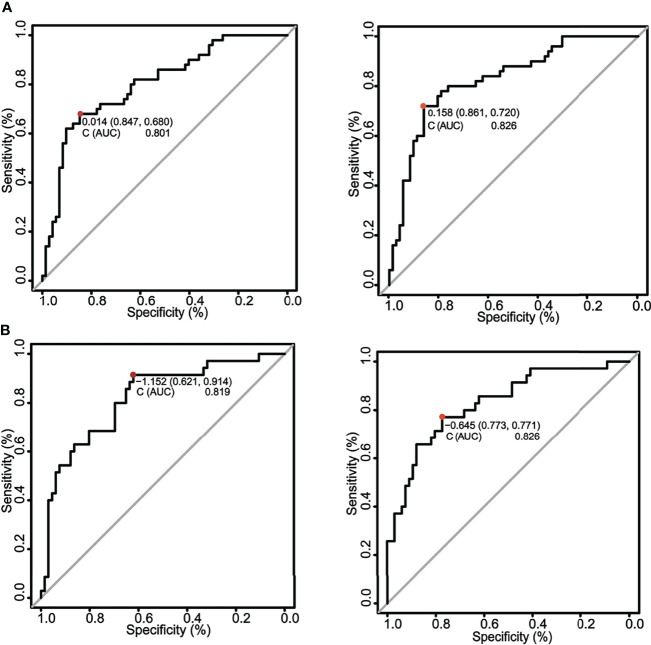
A comparison of ROC curves for the nomograms in training and validation cohorts. **(A)** ROC curves of nomograms in training cohorts. The ROC curves of nomograms with the four risk factors alone (left panel) and an incorporation of MMP-3 in the four risk factors (right panel) in the training cohorts, respectively. The C index of the nomogram generated with the four risk factors alone was 0.801, while the C index of the MMP-3-incorporated nomogram was 0.826. The incorporation of plasma MMP-3 increased the identifying probability of nomogram for ILD in RA patients. **(B)** ROC curves of nomograms in validation cohorts. The ROC curves of nomograms with the four risk factors alone (left panel) and the four factors incorporated with plasma MMP-3 (right panel) in validation cohorts, respectively. The C index of the nomogram generated with the four risk factors alone was 0.819, while the C index of the MMP-3-incorporated nomogram was 0.826. An incorporation of plasma MMP-3 increased the identifying probability of nomogram for the risk of development of ILD in RA patients.

**Figure 7:**
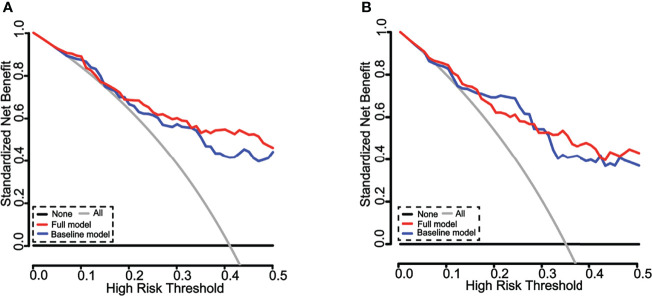
Decision curve analysis (DCA) of the nomograms for estimation of ILD risk in RA patients. **(A)** DCA of the nomograms for identification of ILD in the training cohorts. The x-axis represents the threshold probability, and the y-axis represents the net benefit calculated by adding the true positives and subtracting the false positives. The horizontal line along the x-axis represents the patients without ILD, whereas the gray line represents the assumption that all patients had ILD at a specific threshold probability. The blue line represents the nomogram created with four risk factors alone, and the red line represents the nomogram generated with the four factors with an incorporation of MMP-3. The line better matched to the diagonal gray curve indicates a better prediction. Both of the nomograms could predict the outcome of ILD in RA patients. **(B)** DCA of the nomograms for identification of ILD in validation cohorts. The x-axis represents the threshold probability, and the y-axis represents the net benefit calculated by adding the true positives and subtracting the false positives. The horizontal line along the x-axis represents the assumption of non-ILD RA patients, whereas the gray line represents the assumption that all patients had ILD at a specific threshold probability. The blue line represents the nomogram with four risk factors alone, and the red line represents the nomogram generated with the four factors incorporated with MMP-3. The line better matched to the diagonal gray curve indicates a better prediction. Both of nomograms could predict the outcome of ILD in RA patients.

The changes in C index were used to test the performance of the nomogram with an incorporation of circulating MMP-3 into available clinical risk factors. Indeed, the including of MMP-3 in the combination of four independent risk factors sex, current smoker, RF, and CRP improved the diagnostic accuracy of the nomogram for ILD in RA patients, as compared to that which generated a combination of four risk factors alone (**Figure 6**). The C indexes of the nomogram generated with the four risk factors alone were 0.801 (95% CI, 0.721–0.881) and 0.819 (95% CI, 0.731–0.907) in the training cohorts and validation cohorts, respectively (left panel, **Figures 6A, B**), while the C indexes of the MMP-3-incorporated nomogram were 0.826 (95% CI, 0.751–0.901) and 0.826 (95% CI, 0.740–0.912) in the training cohorts and validation cohorts, respectively (right panel, **Figures 6A, B**). In addition, DCA curves also showed that both of the nomograms could better identify ILD in RA patients, although the net benefits were not significantly improved in both the training and validation cohorts (**Figures 7A, B**).

## Discussion

Although the mortality rate in patients with RA is declining, the death of patients due to RA-ILD is increasing (4), emphasizing the need for improved early diagnosis and intervention in the era of precision medicine. Therefore, we constructed a nomogram to identify the presence of ILD in RA patients. In the present study, we observed that plasma MMP-3 protein was elevated in RA-ILD patients compared with non-ILD RA patients. Five variables (male sex, current smoking, RF, CRP, and MMP-3) were identified by multivariable logistic regression based on univariable analysis and were incorporated into the nomogram for the identification of ILD in RA patients. In consistence, calibration plots graphically exhibited an excellent diagnostic performance by the nomogram. The C-index (0.826 for the training cohorts and 0.826 for the validation cohorts) indicated the satisfactory discriminative ability of the nomogram.

The C index further showed that the nomogram established with a combination of four conventional factors and MMP-3 increased the accuracy in the identification of RA-ILD, as compared to that built with the four risk factors alone. However, DCA curves demonstrated that net benefits were not significantly improved in this study. Of note, DCA cannot replace measures of accuracy such as sensitivity and specificity. This is because DCA mainly focuses on evaluating clinical application, while the ROC is more utilized in evaluating the diagnostic accuracy of the identifying and/or predictive models. In this regard, the area under the ROC curve (AUC) can be interpreted as the probability that a group of individuals experienced the event and the others did not, in which case the individuals who experienced the event had the higher predicted probability (30). Therefore, this observation requires further investigation with more evaluation indicators, such as the net reclassification index (NRI).

MMP-3 is actively associated with joint destruction in RA patients, which has been applied for diagnosing and monitoring the disease activity of RA (17). Additionally, a recent study showed that a higher level of MMP-3 was associated with the interleukin 1β (IL-1β)-induced inflammation response in FLS (31). The above data highlighted the potential clinical value of MMP-3 in the personalized medical management of RA. Previous studies using cell culture models revealed that exposure of lung epithelial cells to MMP-3 led cells to undergo EMT. For instance, when A549 human lung epithelial cells were treated with MMP-3, the expression of EMT signature genes and a breakdown of epithelial cell structure with a conversion to a more spindle-like morphology were altered (32). Moreover, a number of experiments in transgenic mice have suggested that the role of MMP-3 as an inducer from lung type II alveolar epithelial cells leads to the development of fibrotic characteristics (33). Therefore, the aberrant expression of MMP-3 may be instrumental in promoting lung diseases. In agreement with these findings, based on available high-throughput mRNA expression profiles, we uncovered that the expression of MMP-3 was significantly co-up-regulated in RA-FLS and ILD tissues compared to healthy control in the present study. In addition, we found that a higher level of MMP-3 was in plasmas of RA-ILD patients compared with those with non-ILD.

Emerging evidence has shown that baseline characteristics [old age (≥60 years), sex male, smoking, and RA duration] are risk factors for the development of RA-ILD (34–36). Indeed, smokers were prone to develop interstitial lung abnormalities (ILAs), which was associated with numerous pro-inflammatory molecules and MMPs, including MMP-3 (37). This observation was consistent with our identifying result of the nomogram generated from datasets including smoking status and male as risk factors in the development of ILD in RA patients. The present cohorts did not identify old age and RA duration as predominant risk factors in RA-ILD as stated in previous studies (38); we reasoned that it might be due in part to a mild disease status of cohorts in this study.

Apart from the baseline risk factors, several serological indexes, such as RF, C-reactive protein (CRP), and erythrocyte sedimentation rate (ESR), were also demonstrated to have diagnostic values for ILD in RA (9, 39). In agreement with their findings, levels of CRP and RF may be useful biomarkers of risk factors for developing RA-ILD in this study. However, the study showed no association between ESR and RA-ILD in this multivariable model; some patients had elevated ESR probably due to a change in physiological conditions, including strenuous exercises and emotional agitation.

Recently, there is evidence that a proportion of lung disease may be caused by the therapeutic agents used in the treatment of RA, such as methotrexate (MTX). MTX is recommended as the first-line treatment for RA in most regions of the world, and large prospective studies have demonstrated that the use of MTX effectively reduces disease activity and the risk of death in RA patients. However, most of these studies had several potential biases (40, 41). Intriguingly, recent studies and meta-analyses suggest that MTX exposure may be associated with an increased risk in developing ILD in RA patients (42–44). To date, the association between MTX and ILD is still in debate, as no randomized controlled trials in RA-ILD have been conducted in case reports and retrospective studies (14). Similarly, we did not find various disease-modifying antirheumatic drugs to be associated with increased risk of RA-ILD.

Of note, previous studies have reported that antibodies CCP and AKA were risk factors for ILD. However, neither anti-CCP antibody or anti-AKA antibody exhibited a correlation with ILD, and hence they were excluded in single-factor screening in this study. We reasoned that it might be caused by a relatively small size of samples or variant treatments in patients enrolled in this study. This observation requires further investigation with a larger sample size.

There were several limitations in the current study. First, subclinical ILD are frequent extra-articular manifestations of RA; we cannot rule out these patients, mainly due to the lack of a universally accepted evidence-based screening approach. Secondly, although an internal validation of the model yielded optimal discrimination, excellent calibration and bootstrapping are sample reuse methods, and the predictive performance of the nomogram still requires external validation using additional datasets to ensure external applicability, particularly from other research centers. Thirdly, this nomogram model is created by a cross-sectional cohort study and is only able to identify the presence of ILD in RA patients. We further need to collect blood samples before ILD appearance to predict the development of ILD in RA patients. Finally, further follow-up data collection, survival data, and some well-recognized molecular factors could improve this model for future use. The above limitations may be parts of causes of the differences between our study and others. Therefore, the findings presented in this report should be confirmed in subsequent prospective studies.

Collectively, despite these potential shortcomings in this nomogram model, we clearly demonstrate that a combination of conventional risk factors (male sex, current smoking, RF, and CRP) and MMP-3 is strongly associated with the presence of ILD in RA patients. Our findings may facilitate earlier identification of the spectrum of RA-ILD, potentially leading to improvement in clinical outcomes.

## Data Availability Statement

The original contributions presented in the study are included in the article/supplementary material. Further inquiries can be directed to the corresponding authors.

## Ethics Statement

The studies involving human participants were reviewed and approved by The Ethic Committee for the Conduct of Human Research, General Hospital of Ningxia Medical University. The patients/participants provided their written informed consent to participate in this study.

## Author Contributions

JX and JW collected the clinical data. JX, WH, and SW performed the serological analysis. SC collected the plasma samples. JX analyzed the data and drafted the manuscript. XL designed the experiments and revised the manuscript. SC and XL analyzed data and interpreted the data. All authors contributed to the article and approved the submitted version.

## Funding

This work was supported by grants from the National Natural Science Foundation of China (No. 81860132) and grants from the Natural Science Foundation of Ningxia (2019AAC03176 and 2021AAC03369). The funders had no role in the study design, data collection and analysis, decision to publish, or preparation of the manuscript.

## Conflict of Interest

The authors declare that the research was conducted in the absence of any commercial or financial relationships that could be construed as a potential conflict of interest.

## Publisher’s Note

All claims expressed in this article are solely those of the authors and do not necessarily represent those of their affiliated organizations, or those of the publisher, the editors and the reviewers. Any product that may be evaluated in this article, or claim that may be made by its manufacturer, is not guaranteed or endorsed by the publisher.

## Abbreviations

ACPA, anti-citrullinate protein antibodies; ACR, American College of Rheumatology; AEC, alveolar epithelial cell; AKA, anti-keratin antibody; AUC, area under the curve; CENPF, centromere protein F; C-index, concordance index; COPD, chronic obstructive pulmonary disease; CRP, C-reactive protein; DAS28, Disease Activity Score; DCA, decision curve analysis; DIP, desquamative interstitial pneumonia; DLGAP5, disc large-associated protein 5; EMT, epithelial–mesenchymal transition; ESR, erythrocyte sedimentation rate; FC, fold change; FLS, fibroblast-like synoviocytes; HRCT, high-solution computed tomography; ILAs: interstitial lung abnormalities; ILD, interstitial lung disease; IL-1β, interleukin 1β; IPF, idiopathic pulmonary fibrosis; KIF4A, kinesin family member 4A; MMP-3, matrix metalloproteinase-3; MTX, methotrexate; RA, rheumatoid arthritis; RF, rheumatoid factor; ROC, receiver operating characteristic; SD, standard deviation; TOP2A, topoisomerase II alpha.
